# Health system governance for injury care in low- and middle-income countries: a survey of policymakers and policy implementors

**DOI:** 10.1136/bmjgh-2024-017890

**Published:** 2025-02-10

**Authors:** The Equi-Injury Group, Justine Davies, Leila Ghalichi

**Keywords:** Global Health, Health policy, Injury

## Abstract

**Introduction:**

Good health system governance is essential for reducing high mortality and morbidity after injury in low- and middle-income countries (LMICs). Unfortunately, the current state of governance for injury care is not known. This study evaluated governance for injury care in Ghana, Pakistan, Rwanda and South Africa, four LMICs with diverse contexts, to allow understanding of similarities or difference in the status of governance systems in different LMICs.

**Method:**

This cross-sectional study captured the perceptions of 220 respondents (31 policymakers and 189 policy implementers) on injury care governance using the framework for governance in health system developed by Siddiqi. Input was captured in 10 domains: strategic vision; participation and consensus; rule of law; transparency; responsiveness; equity and inclusion; effectiveness and efficiency; accountability; intelligence and information; and ethics.

**Result:**

The median injury care governance score across all domains and countries was 29% (IQR 17–43). The highest median score was achieved in the rule of law (50, 33–67), and the lowest scores were seen in the transparency (0, 0–33), accountability (0, 0–33), and participation and consensus (0, 0–33) domains. Median scores were higher for policymakers (33, 27–48) than for policy implementers (27, 17–42), but the difference was not statistically significant.

**Conclusion:**

The four studied countries have developed some of the foundations of good injury care governance, although many governance domains require more attention. The gap in awareness between policymakers and policy implementers might reflect a delayed or partial implementation of policies or lack of communication between sectors. Ensuring equitable access to injury care across LMICs requires investment in all domains of good injury care governance.

**WHAT IS ALREADY KNOWN ON THIS TOPIC**

- Good healthcare governance is needed to ensure access to quality health systems and reduce avoidable morbidity and mortality. Despite investments in governance in many low- and middle-income countries (LMICs) and a global focus on reducing morbidity and mortality after injury, the current status of injury care governance is not known.

**WHAT THIS STUDY ADDS**

- A survey of policymakers and implementers showed governance scores to be low across contextually diverse LMICs, with some essential governance domains performing better than others. There is a gap in perceptions of existence of governance structures between policymakers and policy implementers.

**HOW THIS STUDY MIGHT AFFECT RESEARCH, PRACTICE OR POLICY**

- Attention and investments are required to improve governance for injury care in all countries. Increasing the awareness of policy implementers of available governance structures and functions is required to improve healthcare services. Evaluations of healthcare governance require assessments conducted with both policymakers and implementers to understand the actions required for implementation of policies to improve patient outcomes.

## Introduction

 Injuries are substantial contributors to the global burden of disease, contributing to 8% of global deaths.^1^ In low- and middle-income countries (LMICs), it is estimated that 8% to 11% of annual disability-adjusted life years are attributable to injuries^2^ and 40% of avoidable deaths after injuries happen due to lack of access to quality care.^3^ Injuries also impose a high workload on the health systems.^4^

Good governance, whereby oversight and management are done in line with the rule of law and to protect human rights, is a central component in improving health system performance.^5^ There are five recognised principles of governance: leadership, integrity, accountability, stewardship and transparency,^6^ that can be further categorised in domains as strategic vision; participation and consensus; rule of law; transparency; responsiveness; equity and inclusion; effectiveness and efficiency; accountability; intelligence and information; and ethics. These principles are central to achieving efficient, equitable and sustainable health systems,^7^ where governance in health systems refers to the structures and mechanisms that oversee the health service functioning. Several frameworks have been developed to assess health systems governance, considering (a) determinants of governance (the existence of procedures, policies, or laws) and (b) governance performance (to what degree laws or procedures have been enforced).

However, despite the global interest in health system governance in the past decade,^8 9^ the need for good health system governance is thought to be particularly acute in LMICs, where there is a large and rapidly evolving burden of disease, resources are limited, and health systems are often not able to keep pace with change. Although many LMICs are investing in improving health system governance, and despite existing evaluation frameworks, governance in health is rarely monitored and evaluated adequately,^10^ whereas in assessing principles of good health system governance, it is possible to identify gaps where improvements can be made.^11^

Governance is essential for injury care. Centralised versus decentralised control impacts the coordination and efficiency of trauma response systems. Ensuring equity addresses disparities in access to emergency care and rehabilitation. Governance also controls technology integration, such as telemedicine and electronic health records which in turn require careful balance of privacy and innovation. Given the high contribution of injuries to the burden of disease, their demands on health systems and the—often—catastrophic health expenditure faced by patients who are injured,^12^ it is imperative that countries consider whether the relevant governance structures exist for injury-related health services to be sustainable, efficient and equitable. In this study, we evaluate governance for injury care in four diverse LMICs, all of which have a high burden of injuries, namely Ghana, Pakistan, Rwanda and South Africa. The different socioeconomic, cultural and injury characteristics of these countries will aid in understanding of whether issues may be experienced similarly across LMIC settings and learning may be transferable between countries. We also explore disparities between the perspective of policymakers and policy implementers regarding governance for health systems for injury care (hereafter, injury care governance).

## Method

### Study settings

The study was conducted in four countries, Ghana, Pakistan, Rwanda and South Africa, all with high injury burden, but diverse socioeconomic, cultural and political contexts; health system structure; and predominant mechanisms of injuries (online supplemental appendix 1).^13^

### Study tool

We used the framework proposed by Siddiqi *et al* to assess governance in health systems.^11^ Siddiqi conceptualised health systems governance in 10 domains: (1) ‘strategic vision’ is about long-term perspective of leaders on health and human development considering historical, cultural and social complexities and a sense of strategic directions for such development; (2) ‘participation and consensus’ focusses on giving everyone a voice in decision-making for health, either directly or indirectly, built on freedom of association and speech and capacity of constructive participation; (3) ‘rule of law’ addresses the fair impartially enforced legal frameworks pertaining to health; (4) ‘transparency’ is the free flow of enough information for understanding and monitoring health matters and direct accessibility of processes, institutions and information; (5) ‘responsiveness’ implies that institutions and processes should try to serve all stakeholders to ensure that the policies and programmes are responsive to the health and non-health needs of the users; (6) ‘equity and inclusion’ require that everyone should have opportunities to improve or maintain their health and well-being; (7) ‘effectiveness and efficiency’ necessitate that the results of the processes and institutions should meet population needs and improve health outcomes while making the best use of resources; (8) ‘accountability’ highlights that decision-makers in government, the private sector and civil society organisations involved in health are accountable to the public and institutional stakeholders; (9) ‘intelligence and information’ are essential for a good understanding of health system, without which it is not possible to provide evidence for informed decisions that influences the behaviour of different interest groups that support, or at least do not conflict with, the strategic vision for health; and (10) ‘ethics’ is about the commonly accepted principles of healthcare ethics such as respect for autonomy, nonmaleficence, beneficence and justice.^11^

The framework was initially developed to explore whole health system governance qualitatively; we adapted it to quantitively evaluate injury care governance and converted it to survey form after a consensus process with academics and policymakers.^14^ This tool was piloted and found to be feasible to administer and have good face validity.^14^ The survey asks questions on the presence or absence of structures or functions at health policymaking and policy implementation levels within the original ten domains. The number of questions per domain ranged from 1 to 12 (online supplemental appendix 2). No translation process was required during this study, given the respondent’s professional language is English.

### Participant selection and data collection

The study was conducted as part of the National Institute for Health and Care Research (NIHR) funded by Global Health Group on Equitable Access to Quality Health Care for Injured People in Four Low- or Middle-Income Countries: Equi-injury.^13^ In each country, we identified health-relevant policymakers (at national and local level) and policy implementers (healthcare managers, doctors, clinical officers, nurses, pre-hospital care providers, physician assistants and other allied healthcare professionals) working in districts where the parent-study was active and invited them to participate. Policy implementers were purposively selected from urban and rural facilities and from different levels of care (primary, secondary and tertiary). Policymakers were selected based on their involvement in health and injury policies. Considering the diverse context of the four countries, different approaches were used to ensure participant enrolment (online supplemental appendix 4). As a result, the distribution of roles of participants recruited varied by country to as detailed in online supplemental appendix 3. Data were collected using an anonymous paper-based or online survey between April 2023 and February 2024. Data were collected using the Research Electronic Data Capture platform.^15^

### Analyses

Data are described as percentages (%), means and SD, or medians and IQR as appropriate. Normality was assessed using Shapiro–Wilk test. For each governance domain, a score was calculated as the percent of maximum achievable scores. The records were included in the analysis if they had more than 95% completeness in the questionnaire. A total injury care governance score was calculated based on the percent of achievable scores from all questions. The injury care governance score is described in total and by domain for all and each country and disaggregated by policy implementer or policymaker. To compare scores across domains within countries, the Kruskal-Wallis test was employed, with Dunn’s post hoc analysis. The Mann-Whitney U test was used for within-country subgroup comparisons between policymakers and policy implementers, given our hypothesis that the awareness and perception of governance components would differ between the policymakers and policy implementers. Very few responses were provided for open-ended questions; thus, a formal analysis was not done. These results did, however, inform the discussion section of the manuscript, whereby the open findings were considered by the authors—many of whom are trauma care providers in the study countries—when understanding the rationale for differences within and between countries.

### Funding

This study is part of the NIHR-funded Global Health Group on Equitable Access to Quality Health Care for Injured People in Four Low- or Middle-Income Countries: Equi-injury.^13^ This research was funded by the NIHR (award number 133135) using UK international development funding from the UK Government to support global health research. The views expressed in this publication are those of the authors and not necessarily those of the NIHR or the UK government.

### Patient and public involvement

The Community Engagement and Involvement is an essential pillar of Equi-Injury project. Community members have been informed and involved from the beginning, with an aim to foster sustainable involvement in research translation to policy.

## Results

Overall, 538 potential participants were approached, and 274 (51%) consented to participate in the study, including 69 from South Africa, 43 from Ghana, 72 from Pakistan and 90 from Rwanda. Participants were included in the analysis if their survey responses were ≥95% complete (220 total, 60 from South Africa, 40 from Ghana, 65 from Pakistan and 55 from Rwanda). (Details of the response rate and included cases are presented in online supplemental appendix 5.) Characteristics of the included respondents are shown in table 1. The majority of them (61%) were male, and 36% were between 31 and 40 years old. Among policy implementers, 114 (60%) were working in urban settings.

**Table 1:** Participant characteristics in injury care governance survey by country

	All countries	South Africa	Ghana	Pakistan	Rwanda
	N (%)	N (%)	N (%)	N (%)	N (%)
Total participants	220	60 (27)	40 (18)	65 (30)	55 (25)
Sex					
Female	83 (37.7)	30 (50.0)	9 (22.5)	22 (33.8)	22 (40.0)
Male	133 (60.5)	29 (48.3)	31 (77.5)	41 (63.1)	32 (58.2)
Other/ prefer not to say	4 (1.8)	1 (1.7)	0 (0.0)	2 (3.1)	1 (1.8)
Age					
21–30	42 (19.1)	4 (6.7)	9 (22.5)	17 (26.2)	12 (21.8)
31–40	79 (35.9)	14 (23.2)	17 (42.5)	29 (44.6)	19 (34.5)
41–50	52 (23.6)	18 (30.0)	10 (25.0)	7 (10.8)	17 (30.9)
51–60	39 (17.7)	21 (35.0)	3 (7.5)	10 (15.4)	5 (9.1)
>60	6 (2.7)	3 (5.0)	0 (0.0)	2 (3.1)	1 (1.8)
Role					
Policy implementer	189 (85.9)	55 (91.7)	35 (87.5)	53 (81.5)	46 (83.6)
Policymaker	31 (14.1)	5 (8.3)	5 (12.5)	12 (18.55)	9 (16.4)
World bank classification	Low- or middle income	Upper middle income	Lower middle income	Lower middle income	Low income

**Figure 1:**
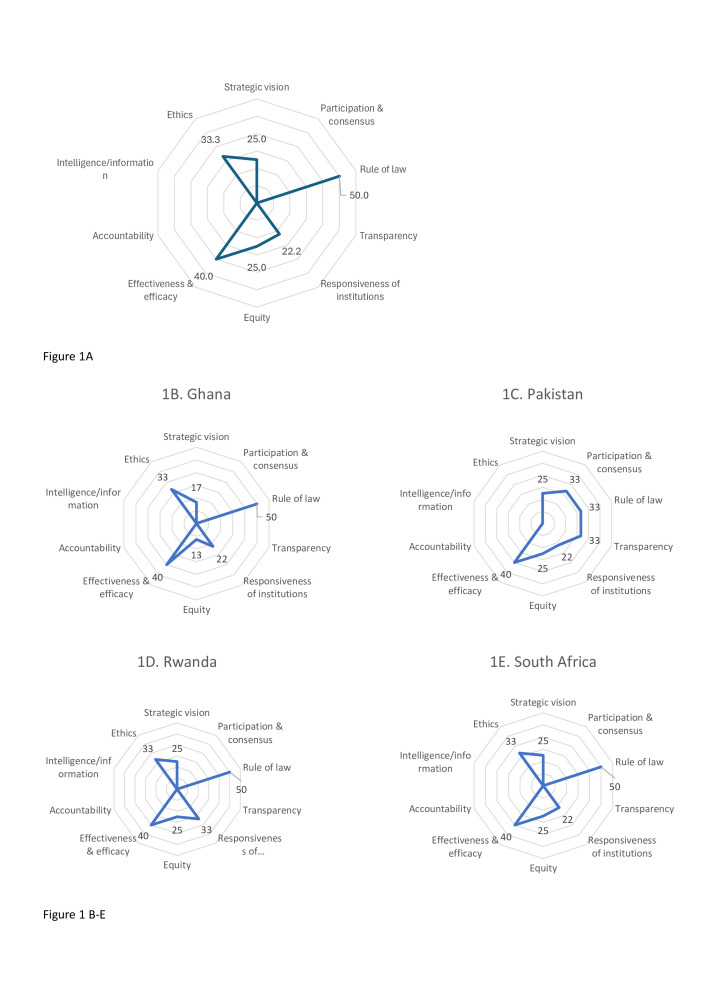
Distribution of governance domain for all countries and participants (**A**) and four countries (**B–E**). Each domain shows percentage of the total achievable score in that domain. Where the median was zero for a domain, a numerical percentage is not shown.

Across all countries, the median injury care governance score was 29% (IQR 17–43): 26% (IQR 16–39) in Ghana, 27% (IQR 17–42) in Pakistan, 31% (IQR 21–48) in Rwanda and 31% (IQR 19–43) in South Africa. In terms of the component domains, combined scores for all countries showed the highest median score was achieved in the rule of law (50%) and the lowest in the transparency, accountability, and participation and consensus domains (all 0%) (figure 1A).

Distribution of total injury care governance scores across individual countries and by domain are presented in table 2. Most countries’ performance was highest in the rule of law and effectiveness and efficacy domains (Figure 1B). In South Africa and Pakistan, ethics, strategic vision, and participation and consensus also outperformed other domains. As for the lowest scoring domains, both participation and consensus and transparency scored lower than other domains in South Africa, Ghana and Rwanda. In Ghana, Accountability was another low-scoring domain. Accountability scored low in Pakistan, along with ethics (table 2).

**Table 2:** The distribution of total injury care governance score and domain scores in all participants and the comparison of policymakers and policy implementors in the four countries, shown as median% (IQR) (the domains are arranged by total score)

		Total		South Africa		Ghana		Pakistan		Rwanda	
Total governance score		29 (17–43)		31 (19–43)		26 (16–39)		27 (17–42)		31 (21–48)	
PI	PM	27 (17–42)	33 (27–48)	29 (19–46)	33 (31–33)	25 (17–37)	27 (8–48)	25 (15–42)	33 (24–47)	30 (17–46)	33 (29–48)
Rule of law		50 (33–67)		50 (25–67)		50 (33–67)		33 (17–50)		50 (33–67)	
PI	PM	50 (17–67)	33 (33–67)	50 (17–67)	50 (50–67)	50 (33–67)	33 (33–67)	33 (17–50)	33 (33–50)	50 (33–67)	50 (33–67)
Effectiveness and efficacy		40 (20–60)		40 (40–60)		40 (20–60)		40 (20–40)		40 (20–40)	
PI	PM	40 (20–60)	40 (20–40)	40 (40–60)	40 (40–40)	40 (20–60)	20 (20–20)	40 (20–40)	20 (20–20)	40 (20–40)	40 (20–60)
Ethics		33 (0–67)		33 (33–67)		33 (0–67)		0 (0–33)		33 (0–67)	
PI	PM	33 (0–67)	0 (0–67)	33 (33–67)	67 (0–67)	33 (0–67)	0 (0–67)	0 (0–33)	0 (0–34)	33 (0–67)	33 (33–33)
Strategic vision		25 (17–33)		25 (17–33)		17 (8–29)		25 (17–42)		25 (17–42)	
PI	PM	25 (17–33)	25 (25–50)	25 (17–33)	25 (25–25)	17 (8–25)	25 (0–50)	25 (17–42)	33 (25–46)	25 (17–42)	25 (25–50)
Equity		25 (13–38)		25 (13–38)		13 (0–38)		25 (13–38)		25 (13–38)	
PI	PM	25 (13–38)	38* (25-38)	25 (13–50)	25 (25–25)	13 (0–38)	38 (0–50)	13 (0–38)	38* (38-38)	25 (0–38)	25 (13–50)
Responsiveness		22 (11–44)		22 (11–44)		22 (6–44)		22 (11–56)		33 (11–56)	
PI	PM	22 (11–56)	22 (11–44)	22 (11–56)	11 (0–22)	22 (11–44)	22 (0–44)	11 (0–56)	22 (11–33)	28 (11–44)	44 (44–56)
Intelligence and information		0 (0–100)		0 (0–100)		0 (0–100)		0 (0–100)		0 (0–100)	
PI	PM	0 (0–100)	0 (0–100)	0 (0–100)	0 (0–0)	0 (0–100)	0 (0–100)	0 (0–100)	50 (0–100)	0 (0–100)	100 (0–100)
Accountability		0 (0–50)		0 (0–50)		0 (0–0)		0 (0–50)		0 (0–50)	
PI	PM	0 (0–50)	50 (0–50)	0 (0–50)	50 (0–100)	0 (0–0)	50 (0–100)	0 (0–50)	0 (0–50)	0 (0–50)	50 (0–50)
Participation and consensus		0 (0–33)		0 (0–33)		0 (0–33)		33 (0–67)		0 (0–33)	
PI	PM	0 (0–33)	33* (0–67)	0 (0–33)	0 (0–66)	0 (0–33)	0 (0–100)	33 (0–33)	67* (33-67)	0 (0–33)	0 (0–33)
Transparency		0 (0–33)		0 (0–33)		0 (0–33)		33 (0–33)		0 (0–33)	
PI	PM	0 (0–33)	33 (0–33)	0 (0–33)	0 (0–0)	0 (0–33)	0 (0–33)	33 (0–33)	33 (17–67)	0 (0–33)	33 (0–33)

Green and red cells show statistically significantly higher and lower scores, respectively, compared with other domains within each country.

* Indicates a significant difference between policymakers and policy implementor group (p<0.05).

PIpolicy implementorPMpolicymaker

Numerical scores were higher for policymakers than policy implementers; however, the difference was only statistically significant for the domains of equity and participation and consensus for all countries together and Pakistan alone (table 2).

.

The scores of urban and rural policy implementers are presented in online supplemental appendix 6. In summary, the scores were largely the same, with some differences particularly in Pakistan, where rural participants scored numerically higher in some domains.

## Discussion

We have evaluated the perceptions of policymakers and policy implementers on injury care governance structures and functions across four contextually diverse LMIC settings. The total injury care governance scores were similar in the four LMICs. The scores reflect the respondents’ awareness or perceptions on governance structures or functions for injury care and not the actual state of governance. However, for good governance to be achieved, policymakers and implementers need to be aware of the structures and processes to enable it. The scores therefore indicate that across all countries, there is room for improvement in either developing or improving the awareness of existing structures and processes for healthcare systems for injury.

We found a distribution of injury care governance scores across domains which could indicate areas of priority for each country. However, despite differences between countries’ contexts and injury burden, similar patterns emerged when evaluating the individual domains; similarities were more striking in the three African countries.

All countries scored highest in the domain of *rule of law*, potentially reflective of laws to protect against road traffic trauma, including for wearing seat belts and helmets, road speed limits; and standards for workplace safety.^16^ Legislation also exists mandating that all public and private hospitals provide emergency care for injured patients irrespective of their financial and insurance status in all countries.^17 18^ However, in practice, there are gaps in enforcement, and the focus is often on ethical and professional responsibilities, despite existing rules. The question on accreditation of injury care providers in the domain of rule of law scored numerically lower than others in this domain. Although all countries have some form of accreditation process in place, carried out by different organisations, their implementation is thought to be limited and highly variable.^19^

All countries scored higher in *effectiveness and efficacy* compared with other domains. Most countries have prehospital systems available and many have been strengthened in recent years but shortages in prehospital services are frequently experienced.^20^ Across all countries public and local transport might be used along with the prehospital referral systems.^21 22^ Availability of guidelines also may contribute to *effectiveness and efficacy*; however, Ghana is the only country in our study with guidelines for injury care developed by the Ministry of Health (MoH).^17^

The domain of *ethics* received a greater score in the three African countries than in Pakistan, where research and practice ethics are practiced and enforced within teaching and private medical institutes and not at national level.^23^ *Participation and consensus* and *transparency* received significantly lower scores than other domains in Ghana and South Africa, with Pakistan achieving the highest score in this domain, potentially reflective of the involvement of stakeholders such as non-governmental organisations and private companies in health policy formulation for trauma.^24 25^ In South Africa, although there are numerous active community groups with mandates embedded within statutory services, these are less prominent in formulation of policy at national and provincial levels.^26^,^28^ In fact, in South Africa, the participatory governance mandated with formal structures and processes in public sector has limited functionality in practice, and this could have influenced the finding.^29 30^ In Ghana, whereas policy documents suggest engaging with stakeholders is encouraged,^31^ the low scores allocated both by policymakers and policy implementers might reflect lack of comprehensive implementation of such policies beyond managers or senior clinicians.

Across all countries, *accountability* and *intelligence and information* were low-scoring domains, despite author-recognised efforts in partner countries. In South Africa, specific mechanisms govern the reporting process of failed trauma services to policymakers or authorities. In Rwanda, the MoH has a dedicated trauma and disability unit which oversees the country’s injury care system. This unit has set annual performance goals and is responsible for assessing healthcare facilities, although our work suggests that many respondents are not aware of such provisions. Institution-level attempts have been made to establish internal audit and performance review systems to improve accountability in Ghana and Pakistan. In some cases, further steps have been taken to address the identified gaps.^32 33^ Occasionally, there appear to be periodic and irregular mechanisms for reporting failing trauma systems and correcting the underperformance of trauma systems based on events rather than streamlining systematic mechanisms. In Ghana, some information on financial and managerial aspects of injury care is collected, but it appears that there is low availability and use of this data in decision-making.^34^

Regarding *intelligence and information*, there are no national trauma registries to capture data on injured patients in Pakistan or South Africa, whereas in Ghana and Rwanda, there have been efforts to develop these.^35^,^37^ In Pakistan, there has been a pilot testing for trauma registry implementation in a private sector institute.^38^ Information about clinical care data collection appears not widely known among our study respondents, and injury care patient satisfaction outcomes are not routinely recorded in any country, apart from for research studies.^39 40^ General mandatory injury and post-injury data collected is not widely practiced or frequently used in policymaking in our studied countries. Therefore, although the domain of *responsiveness* scored reasonably well, without good intelligence and information, being responsive to experienced needs will be challenging.

Given that it is expected that those involved in caring for injured patients would be aware of the ongoing and implemented strategies, policies and services, our finding that policy implementers generally scored lower than policymakers in the domains is an important signal of weakly implemented policy. This pattern also indicates the delayed transition of governance from a policymaking to an implementation and establishment phase. It also possibly signals a lack of policy implementers’ engagement in the process of policy formulation, leaving out some important stakeholders with influence and agency over realising policy commitments and intent around good governance in injury care. Poor implementation might also result from a lack of necessary infrastructure and, thus, the absence of governance structures and functions, despite their inclusion in policy. The existing gap between policy and implementation along with diverse levels of decentralisation across all four countries is a common issue in our study setting.

Although there was some divergence in results across countries, the findings were remarkably similar across study settings, despite diverse cultures and contexts. Considering the diverse context of the four countries, these findings are therefore likely to be similar to those in other LMICs.^41 42^ The proportion of the public health budget dedicated to injury care may also be affected by other competing priorities such as infectious diseases, maternal health and child health programmes, which remain prominent in the Sustainable Development Goals.^43^It is also affected by countries’ total health budget, percent of gross domestic product spent on health, and the interests and influence of donors and development partners.^44 45^

For those countries wishing to improve health systems for injury care, like our study countries, many face sparsity of data to evidence health outcomes after injury,^46 47^ whereas this information is essential for the planning and improvement of healthcare services for injured people. That World Health Assembly resolution 76.2 focuses on emergency, critical and operative care may help to galvanise countries towards both data collection and evidence-informed health system provision for injured patients and related governance structures and functions. Our study shows areas where improvements in governance structures and functions are needed.^48^

Actioning improvement in injury care governance will require balancing the interests of various actors and factors. For example, an adapted ‘governance triangle’ has been proposed which considers six governance spaces within and between key stakeholder categories of policymakers, service providers and people.^49^ Institutionalisation is a further important concept, addressing the ‘process of transforming ideas into programmes and automating actions’ and ‘meaningful involvement of leaders, politicians, civil society and public participation’.^8^ Institutionalisation ensures that governance principles become integral and enduring parts of the institution’s operations, which standardises and stabilises governance practices, creating formal rules and procedures to guide behaviour and decision-making, fostering stability and resilience over time.^8^ Contemporary debates have also focused on decentralisation, equity in access and outcomes, technology and privacy, and public trust amid misinformation. The role of the private sector, tensions between global health initiatives and local governance, and accountability and transparency in complex, multi-layered health systems are further considerations.^45^ However, ideas and frameworks around governance point to the importance of addressing each principle of governance as well as appreciating their interactions and inter-dependencies, and ensuring all key stakeholders are represented in processes to ensure governance structures are built, executed and sustained.

The findings underscore an urgent need for comprehensive reforms that bridge the policy/implementation gap in injury care governance. Practical implications include measures such as and including accountability mechanisms, enhanced stakeholder engagement and clear operational frameworks enacting governance principles. Strengthening data systems, such as trauma registries and formally integrating them into decision-making are further critical measures to improve responsiveness and outcomes. Moreover, capacity-building for implementers, robust monitoring and evaluation, and mechanisms for continuous feedback between policymakers and implementers are essential. Decentralising governance processes and fostering participatory governance by including local actors and community groups could also enhance functionality and sustainability of injury care systems.

Other studies of healthcare governance in LMICs have reported comparable challenges, such as weak enforcement of health policies, fragmented accountability mechanisms and limited stakeholder participation.^29 30 34^ This study aligns with the global governance literature in identifying the need for institutionalisation and operationalisation of governance principles and underscores the importance of contextualised approaches.^50^ Future studies should investigate the impact of specific reforms on injury care governance and explore strategies for participation and monitoring. Additionally, further cross-contextual comparative analyses would strengthen the generalisability of findings.

There has been no survey assessing governance for injury care in high income countries, of which we are aware. However, when appraising individual aspects of governance, high-income countries (HICs) generally have several established injury care governance structures and processes which may be absent or nascent in LMICs. Injury care is addressed in several national policies, although the implementation varies across regions. Trauma centres’ audit, and access and adherence to guidelines are all measures to achieve optimal care for trauma patients in many HICs.^51^ Most HICs collect data, often in the form of trauma registries, to optimise trauma care and guide national strategies.^52^,^54^ Using the example of the UK, implementing trauma systems is a key component of efforts to achieve an equitable and resilient healthcare system;^55 56^ the National Major Trauma Registry monitors the outcomes and processes for trauma care to enhance patient safety and outcomes through data-driven strategies; trauma nursing education is addressed during continuing professional development courses,^57^ while emergency medical doctors are trained in a nationally accredited training programme;^58^ ethical and legal principles and directives guide both treatment and research involving trauma patients; and medical professionals are regulated by professional organisations (eg, the General Medical Council for doctors); patients are also encouraged to input into decision-making, generally by a semiformal process of engaging with their local parliamentary representatives. However, the complexity and diversity of the field necessitates ongoing efforts to address existing and emerging challenges^59^ and additional strategies, policies and fundings are recognised to be required to improve organisational culture and practices to address the impact of trauma on patients.^60 61^

## Limitations

Our study did not assess whether responses were correct; however, our intention was not to determine the actual injury care governance in each country, but rather the awareness and perceptions of participants of governance structures and functions for injury care. Given that to action governance structures and functions requires awareness of them, we believe that assessing awareness or perceptions of these is essential. Several frameworks have been developed to assess governance.^1144 62^,^64^ We elected to use that of Siddiqi *et al*, given our previous experience of validating this in LMIC settings.^14^ We did not apply any weighting while comparing the domains. This was due to a lack of theoretical background on the proportional weight of the domains in determining the outcome. We tried to approach a diverse population of policymakers and policy implementers, but the response rate was different in subgroups. Where possible, explanations of reasons behind results, as discussed by authors, are referenced; however, given that policymaking and implementing is often understudied and lack of action is not often captured in official documents, not all statements are backed up by official references. Finally, most local policymakers and all policy implementers from Pakistan’s health system were recruited from Sindh province. Thus, findings from this study may have more limited generalisability to the rest of the country. Also, Pakistan was the only country with some policy implementers recruitment from a private hospital.

In conclusion, the four countries in this study have a foundation on which to build good injury care governance, although some governance domains require more attention than others. The gap in awareness between policymakers and policy implementers suggests that across most governance domains, structures and functions have been developed on paper, but not implemented. That despite varying contexts, the injury care governance scores and the discrepancies between policy implementers and policymakers were quite similar, suggests that our findings may apply across more settings than were represented in our study and that improving access to equitable care after injuries across LMICs requires investment in health systems and its requisite governance structures and functions.

## supplementary material

10.1136/bmjgh-2024-017890 online supplemental file 1

## Data Availability

Data are available upon reasonable request.
