# Supplementary material for: Health system governance for injury care in low- and middle-income countries: a survey of policymakers and policy implementors
Source: BMJ Glob Health. 2025 Feb 10;10(2):e017890. doi: 10.1136/bmjgh-2024-017890 (PMC11815436; doi:10.1136/bmjgh-2024-017890)
Supplement: online supplemental file 1 [file bmjgh-10-2-s001.pdf]

Appendix 1. Partner countries' economic and health expenditure profile at the time of study, based on information from the World Bank.

	South Africa	Ghana	Pakistan	Rwanda
**Income Status**	Upper-middle	Lower-middle	Lower-middle	Low
**HDI rank** (1)	110	145	164	161
**GINI index** (2)	63	43.5	29.6	43.7
**% GDP on health**	8.3	4.2	2.9	7.3
**External HE (% current)**	1.4	8.9	10.3	35.9
**Private HE (% current)**	38.2	37.1	60.7	23.3
**OOP HE (% current)**	5.5	27.2	57.5	10.1

HE: Health expenditure; HDI: Human Development Index; OOP: Out of Pocket; GDP: Gross Domestic Product. Colouring for each variable show the differences between countries (darker colour represents higher numerical value)

(1) HDI report 2022
(2) WB report, accessed May 2024

Appendix 2.

Instrument for Governance survey, and its domains.

**Domain**	question
**Strategic vision** (Maximum achievable score: 12)	1. Is there specific mention of trauma in the national health plan or are there any specific national health policies around trauma care?
	2. Is there a national trauma strategy?
	3. What percentage of the country's public health budget goes to providing trauma care in the public sector?
	4. Is public sector trauma care provided free of charge?
	5. Is there a national insurance scheme that covers trauma care?
	6. Is there a department within the National Ministry of Health dedicated for Trauma?
	7. If the National health plan mentions trauma, or if there are national health policies for trauma care, are there specific objectives relating to trauma and timelines to achieve them?
	8. If you have answered yes to previous question, have any of those specific trauma objectives been implemented?
**Participation & consensus** (Maximum achievable score: 3)	9. Are stakeholders (e.g.: NGOs, private companies) involved in health policy formulation for trauma?
	10. What is the level of stakeholder engagement/community participation at the national and provincial level in implementation of trauma interventions?

**Rule of law** (Maximum achievable score: 6)	11. Is there accreditation of trauma care providers (doctors, nurses, etc) and are these enforced?
	12. Are there laws to enforce a duty of care by hospitals to treat uninsured trauma patients?
	13. Are there laws to protect against trauma and are these laws enforced? Example Seat belt laws?
**Transparency** (Maximum achievable score: 3)	14. Is information readily available on the financial commitment to or allocated budget for trauma care in the public sector at either national or provincial level?
	15. Are managers (District Directors of Health, Medical Superintendents of hospitals) evaluated on their health facility or facilities reaching specific targets for trauma care? And if so, are the results of these evaluations available and accessible?
**Responsiveness of institutions** (Maximum achievable score: 9)	16. Is there mandatory reporting of health facility trauma data?
	17. Is there a national trauma registry (information management for trauma care)? Is it used? In both private and public?
	18. Are these data used to inform national or provincial policy?
	19. Are data on clinical post-injury outcomes captured routinely by health facilities?
	20. Are data on injury care patient satisfaction routinely captured?
	21. Are injury care patient satisfaction data used in planning services?
	22. What is the level of responsiveness of the health system to non-medical needs of injured people (social needs, mental health needs etc)?
**Equity** (Maximum achievable score: 8)	23. Are there national level financial schemes to ensure the poor who are injured do not have to pay out of pocket direct medical costs of trauma care?
	24. Are there health policies in place to address inequality in access to care for trauma?
	25. Are there data which show whether access to care at a local or national level is equitable or not? And do those data show that access is equitable?
	26. Is the allocation of trauma care staff to districts and hospitals appropriate and based on needs?
	27. Is there a mechanism to equitably distribute the budget for health or trauma care?
**Effectiveness & Efficacy** (Maximum achievable score: 5)	28. Are there national or provincial guidelines for in -service training of staff on trauma care?
	29. Is there a pre-hospital referral system (e.g.: ambulance service)?
	30. If pre-hospital referral systems are available, what has been the experience of patients regarding pre-hospital referrals?
	31. Are local transport operators (e.g.: taxis) involved in transporting people to hospital?
**Accountability** (Maximum achievable score: 2)	32. Are there mechanisms to report failing trauma services to policy makers or regulatory authorities?
	33. Are there any mechanisms of correcting under performance of trauma services?
**Intelligence & Information** (Maximum achievable score: 1)	34. Do staff providing trauma services understand what data needs to be captured to improve trauma services and do they have the right data capturing tools to enable them to do this?
**Ethics** (Maximum achievable score: 3)	35. Is there any policy available for regulating trauma related research?
	36. Are there any standard operating procedures in place to ensure quality and ethical trauma care for injured people?
	37. Are there mechanisms in place in institutes for enforcing high ethical standards in the treatment of trauma patients?

Appendix 3. Type of participants in the Governance survey

	South Africa	Ghana	Pakistan	Rwanda	All countrie s
**Policy maker national government**	2 (3%)	2 (5%)	7 (11%)	4 (7%)	15 (7%)
**Policy maker local government**	3 (5%)	3 (8%)	5 (7%)	5 (9%)	16 (7%)
**Healthcare manager**	8 (13%)	3 (8%)	5 (7%)	5 (9%)	21 (10%)
**Healthcare provider - doctor**	25 (42%)	9 (23%)	20 (31%)	10 (18%)	64 (29%)
**Healthcare provider - clinical officer**	2 (3%)	2 (5%)	2 (3%)	5 (9%)	11 (5%)
**Healthcare provider - nurse**	14 (23%)	16 (40%)	22 (34%)	18 (33%)	70 (32%)
**Healthcare provider - prehospital care**	2 (3%)	2 (5%)	3 (5%)	2 (4%)	9 (4%)
**other**	4 (8%)	3 (8%)	1 (2%)	6 (11%)	14 (6%)

Appendix 4. Data collection process in the countries:

In South Africa, participants were selected through purposive snowball sampling of policy implementors and policy makers known to the research team. A total of 96 potential participants were approached by introductory email, at the hospital study sites, as well as at workshops or individual in-depth interviews on injury policymaking. Rural and urban sites recruited in the same manner. During the workshops, policy implementors were given a link to the survey to complete. Participants who did not attended the workshops completed the survey through an email link.

In Ghana, participants were selected through purposive sampling of policy implementers and policy makers based on their known involvement in injury care. Forty-three participants were approached, and all agreed to participate. The participants were approached during the individual and multistakeholder workshops on their involvement in injury policymaking held at the rural and urban study sites. Before the start of the workshops, the participant information leaflet regarding the survey was given to them to read, after which informed consent was taken. The participants then completed printed copies of the survey which were subsequently transferred to Redcap by the research team. The two national level policymakers who did not attend any of the workshops were contacted via telephone to invite them to participate. Subsequently, they were given the Redcap link to the survey to complete.

In Pakistan participants were selected through purposive snowball sampling of policy implementers and policy makers based on their known involvement in injury care. Twenty policy makers and fifty-eight policy implementers were approached. The governance survey was administered during the policy implementers individual workshops on their involvement in injury policymaking held at the rural and urban study sites while policy makers were approached during their in-depth interviews. Before

the start of the workshops, informed consent was taken. The participants then completed printed copies of the survey which were subsequently transferred onto Redcap by the research team.

In Rwanda, a combination of online and paper surveys was used. The online survey link was emailed directly to four facility managers, who then distributed the link to an estimated 70 healthcare providers under their supervision. Additionally, the link was sent to 21 local and national policymakers. For policymakers who could not use the link, a paper version of the survey was distributed. Specifically, 20 paper surveys were delivered to policymakers at the Rwanda Biomedical Center and the Ministry of Health (MoH). In total, 41 policy makers and 280 policy implementers were invited to participate.

Appendix 5. The response rate of the participants and percent of eligible records in total and in four countries.

	**Response rate**	**Percent of eligible records**
**Total**	51%	80%
**South Africa**	72%	87%
**Ghana**	100%	93%
**Pakistan**	92%	90%
**Rwanda**	28%	61%

* Records were eligible for inclusion in the analysis if at least 95% of the survey questions were answered.

Appendix 6. The distribution of total injury care governance score and d*omain scores* in the four countries, shown as Median (IQR).

	**Total**		**South Africa**		**Ghana**		**Pakistan**		**Rwanda**	
	Urban<br>N=114	Rural<br>N=74	Urban<br>N=45	Rural<br>N=10	Urban<br>N=23	Rural<br>N=11	Urban<br>N=31	Rural<br>N=22	Urban<br>N=14	Rural<br>N=30
**Governance**	**26**<br>(17-42)	**27**<br>(17-44)	**29**<br>(19-44)	**37**<br>(19-48)	**27**<br>(19-42)	**23**<br>(7-37)	**21**<br>(14-30)	**40**<br>(17-52)	**40**<br>(25-50)	**25**<br>(15-44)
Rule of law	50<br>(17-67)	42<br>(33-67)	50<br>(17-67)	50<br>(50-83)	50<br>(33-67)	33<br>(17-67)	33<br>(17-50)	33<br>(17-50)	58<br>(33-100)	42<br>(33-67)
Effectiveness & efficacy	40<br>(20-60)	40<br>(20-40)	40<br>(40-60)	40<br>(40-60)	40<br>(20-60)	40<br>(20-60)	30<br>(20-40)	40<br>(20-40)	40<br>(20-40)	40<br>(20-40)
Ethics	33<br>(0-67)	0<br>(0-67)	33<br>(33-67)	67<br>(67-67)	33<br>(0-67)	33<br>(0-67)	0<br>(0-33)	0<br>(0-0)	50<br>(0-67)	33<br>(0-67)
Strategic vision	25<br>(17-33)	25<br>(17-33)	25<br>(17-33)	25<br>(17-33)	17<br>(8-25)	17<br>(8-33)	21<br>(17-33)	33<br>(17-75)	42<br>(25-50)	25<br>(17-33)
Equity	25<br>(13-38)	25<br>(13-38)	25<br>(13-50)	25<br>(25-50)	25<br>(13-38)	13<br>(0-25)	13<br>(0-25)	38<br>(13-50)	25<br>(0-50)	25<br>(0-38)
Responsiveness	22<br>(11-56)	22<br>(11-56)	22<br>(11-56)	17<br>(0-44)	33<br>(11-56)	11<br>(0-33)	11<br>(0-17)	44<br>(11-67)	33<br>(11-44)	11<br>(11-56)
Intelligence & information	0<br>(0-100)	0<br>(0-100)	0<br>(0-100)	0<br>(0-100)	0<br>(0-100)	0<br>(0-0)	0<br>(0-0)	100<br>(0-100)	100<br>(0-100)	100<br>(0-100)
Accountability	0<br>(0-50)	50<br>(0-50)	0<br>(0-50)	50<br>(0-100)	0<br>(0-0)	0<br>(0-50)	0<br>(0-0)	50<br>(0-100)	25<br>(0-100)	0<br>(0-50)
Participation & consensus	0<br>(0-33)	33<br>(0-67)	0<br>(0-33)	0<br>(0-33)	0<br>(0-33)	0<br>(0-33)	33<br>(0-50)	33<br>(0-33)	33<br>(0-67)	0<br>(0-0)
Transparency	0<br>(0-33)	33<br>(0-33)	0<br>(0-33)	0<br>(0-67)	0<br>(0-33)	0<br>(0-33)	33<br>(0-33)	33<br>(0-67)	0<br>(0-67)	0<br>(0-67)
